# Atherosclerotic vascular disease is more prevalent among black ESKD patients on long-term CAPD in South Africa

**DOI:** 10.1186/s12882-019-1583-8

**Published:** 2019-10-30

**Authors:** S. O. Oguntola, M. O. Hassan, R. Duarte, A. Vachiat, P. Manga, S. Naicker

**Affiliations:** 1 Department of Internal Medicine, Division of Nephrology, Faculty of Health Sciences, University of Witwatersrand, 7, York Street, Parktown, Johannesburg, South Africa; 2 Department of Internal Medicine, Division of Cardiology, Faculty of Health Sciences, University of Witwatersrand, Johannesburg, South Africa; 3 Department of Internal Medicine Laboratory, Faculty of Health Sciences, University of Witwatersrand, Johannesburg, South Africa; 4 Department of Internal Medicine, Ladoke Akintola University of Technology Teaching Hospital, Osogbo, Osun-State Nigeria

**Keywords:** Atherosclerotic vascular disease, Continous ambulatory peritoneal dialysis, Haemodialysis, Chronic kidney disease, End-stage kidney disease, Left ventricular hypertrophy

## Abstract

**Background:**

Occurrence of cardiovascular disease (CVD) in the setting of chronic kidney disease (CKD) can be described as a “cruel alliance”, with CVD responsible for about half of all deaths among CKD patients. Chronic kidney disease patients are more likely to die from CVD than progress to end stage kidney disease (ESKD). Dyslipidaemia, a known traditional risk factor for CVD, is highly prevalent among CKD patients and with an even higher frequency among ESKD patients on dialytic therapies. Prolonged exposure of continuous ambulatory peritoneal dialysis (CAPD) patients to high glucose concentrations in CAPD fluid have been associated with increased risk of cardiovascular events. In this study, we investigated the relationship of atherosclerotic vascular disease (AsVD) to clinical and echocardiographic parameters among black South Africans with CKD (stage 3) and ESKD on CAPD and haemodialysis (HD).

**Methods:**

This was a cross-sectional study of 40 adult (18–65 years) non-diabetic CKD patients (kidney disease outcome quality initiative [KDOQI] stage 3), 40 ESKD patients on CAPD, 40 ESKD patients on HD and 41 age and sex-matched healthy controls. An interviewer-administered questionnaire was used to obtain information on participants’ sociodemographic and cardiovascular risk factors. Anthropometric parameters were measured. Serum blood samples were analysed for creatinine, albumin and lipid profile; lipoprotein ratios, Framingham’s risk score and the 10-year risk of developing coronary heart disease (CHD) were calculated. Echocardiography was performed on all patients and carotid intima media thickness (CIMT) was measured in both right and left carotid arteries at 1 cm proximal to the carotid bulb. Spearman’s rank correlation and binary logistic regression were conducted to determine the relationship of AsVD to clinical and echocardiographic parameters.

**Results:**

Atherosclerotic vascular disease was most prevalent among ESKD patients on CAPD (70%, *n* = 28/40). Chronic kidney disease and HD patients exhibited a similar prevalence (47.5%, *n* = 19/40), while the prevalence in controls was 17.1% (*n* = 7/41). Presence of AsVD was associated with significantly older age, higher waist hip ratio (WHR), left ventricular mass index (LVMI) and Framingham’s 10-year risk of developing CHD. Significant differences in clinical and echocardiographic parameters were observed when the study groups were compared. Age and LVH independently predicted AsVD.

**Conclusion:**

Atherosclerotic vascular disease was more prevalent among CAPD patients compared to pre-dialysis CKD and HD patients. Among all lipoprotein ratios assessed, non-HDL-C showed the most consistent significant difference between the groups. Age (> 40 years) and presence of LVH were independent predictors of AsVD.

## Introduction

Robust association exists between chronic kidney disease (CKD) and cardiovascular disease (CVD) [1], of which, atherosclerotic vascular disease (AsVD) contributes significantly to morbidity and mortality in CKD [2]. Atherosclerotic vascular disease was found to be related to 60.9% of deaths among non-diabetic end stage kidney disease (ESKD) patients on maintenance haemodialysis (HD) [3]. As the estimated glomerular filtration rate (eGFR) deteriorates, cardiovascular complications emerge and increase in frequency as CKD progresses [4]. A report of the baseline characteristics of participants from the Chronic Renal Insufficiency Cohort (CRIC) study showed that lower levels of eGFR were associated with a higher prevalence of CVD [5]. Similarly, the Atherosclerotic Risk In Community (ARIC) study found a consistent association between increased left ventricular mass and low eGFR [6]. In the sub-Saharan African setting, a study found cardiac lesions to be highly prevalent among ESKD patients on maintenance HD, with left ventricular hypertrophy (LVH) being the most common cardiac lesion [7]. A case control study among black South African ESKD patients found a high prevalence of carotid plaques (38.1%) among ESKD patients on maintenance HD compared to controls (7.9%) [8]. Similarly, coronary calcification was present in 38.6% of ESKD patients in another South African study and was associated with older age and previous CVD [9]. Although this study recruited both HD and CAPD patients, nearly 90% of the participants were on HD [9]. Studies designed to assess CVD in black South African ESKD patients on CAPD are sparse, despite increased CVD-related morbidity and mortality seen in this group of patients [10, 11]. A study on the predictors of mortality among rural dwelling ESKD patients on chronic dialysis found continuous ambulatory peritoneal dialysis (CAPD) to be a predictor of all-cause mortality [11], hence there is the need to investigate CVD in both HD and CAPD patients.

Dyslipidaemia is recognized as a traditional risk factor for CVD. Furthermore, abnormalities in lipid metabolism related to uraemia have been reported [12]. A rise in the prevalence of atherogenic dyslipidaemia in the black population in an urban settlement in Cape Town, South Africa has been noted; 59% of ischaemic heart disease and 29% of ischaemic stroke burden in males and females ≥30 years were attributable to increased cholesterol levels in this population [13]. Some studies have reported that dyslipidaemia, such as an increase in low density lipoprotein (LDL-C), triglycerides (TG), lipoprotein (a) (LPa) and reduced high density lipoprotein (HDL-C) are more commonly seen in PD than HD patients [14, 15].

The high prevalence of dyslipidaemia in the urban population of South Africa, the association between dyslipidaemia and CKD and the evidence of higher mortality in PD when compared to HD, impelled us to evaluate the prevalence and the determinants of atherosclerotic vascular disease in CKD (stage 3) and ESKD patients on dialysis (both CAPD and HD).

## Methods

This was a cross-sectional study of 40 adult (age 18–65 years) non-diabetic ESKD patients on maintenance HD, 40 ESKD patients on CAPD, 40 stage 3 CKD patients and 41 age- and sex-matched healthy controls at a large urban public hospital in South Africa from 2 January 2017 to 31 August 2017. The CAPD patients were on 4 exchanges per day of 2 l bags of conventional (glucose-based) PD fluid, while the HD patients were on thrice weekly maintenance dialysis on bicarbonate dialysate using a high flux dialyzer with biocompatible membranes, dialysate flow rate of 500 ml/min and moderate blood flow rate of 350 ml/min. Black patients were selected as the study cohort as they formed the predominant dialysis group, comprising over 95% of the dialysis population at our centre. Diabetes mellitus was an exclusion in this study, so as to exclude the effects of diabetes on atherosclerosis. The study was approved by the Human Research Ethics Committee (HREC) of the University of the Witwatersrand, study number M160614. An interviewer-administered questionnaire was used to obtain information on participants’ socio-demographic and cardiovascular risk factors including age, gender, waist-hip ratio (WHR), body mass index (BMI).

Waist and hip circumference and WHR were measured with patients in an erect position and after PD fluid had been drained out in CAPD patients. Weight was measured by using a weighing scale. In HD patients, the weight was obtained before initiation of dialysis and at the end of dialysis, however, the post-HD weight was used in analysis; weight was obtained in CAPD patients after the PD fluid was drained. Height was assessed by a stadiometer. Body mass index was calculated using the formula weight/height [2], while the body surface area was calculated using the Mosteller formula [16]. Three blood pressure readings were taken on the left arm with an appropriate sized cuff, 10 min apart, after the patient had rested for 5 min in a sitting position. The average of the last two readings was recorded as the blood pressure. Blood samples were taken before dialysis in HD patients. A serum lipogram (total cholesterol [TC], LDL-C, TG and HDL-C) was determined by an enzymatic colorimetric method using Roche cobas 8000 modular analyser series, module c701 analyzer (Roche, Japan). Lipoprotein ratios were calculated as follows: Atherogenic index of plasma (AIP) = Log (Tg/HDL-C); Castelli index 1 = TC/HDL-C; Castelli index 2 = LDL-C/HDL-C; non-HDL-C = TC-HDL-C; Atherogenic index (AI) = non-HDL-C/HDL-C; Lipoprotein combined index (LCI) = (TC × TG × LDL-C)/HDL-C [17]. Estimated GFR was calculated in controls and stage 3 CKD patients using the 2009 CKD-EPI formula [18]. All participants had echocardiography and CIMT measurements in accordance with the guidelines of the American Society of Echocardiography [19], using a Philips iE33 echocardiography machine (Philips Corporation, USA). The CIMT measurement was done before initiation of HD in HD patients and after draining the abdomen in CAPD patients. Carotid intima media thickness was assessed using the vascular probe of the echocardiography machine, Philips iE33 (S5–1 probe) by focussing on the far wall of the carotid artery, 1 cm proximal to the dilatation of the carotid bulb along the long axis of the artery. Automatic echo-generated measurements with percentage quality of 95% were recorded. The procedure was carried out on both left and right carotid arteries and the average used in the analysis. Atherosclerotic vascular disease was defined by using a combination of increased CIMT (> 0.55 mm) and presence of plaques.

### Power calculation and data analysis

Stata version13.1 (StataCorp, USA) and IBM SPSS version 20 (IBM Corporation, Armonk, New York) were used for statistical analysis. The power to detect differences in levels of atherosclerosis was calculated. With a sample size of 40 in each group, a 5% significance level and atherosclerosis proportion of 38.1% in haemodialysis patients compared to 7.93% in controls [8], there will be a power of 90.89 to detect differences in these groups.

Categorical variables were expressed as frequencies and percentages. Test of normality (Shapiro-Wilk) was performed on all continuous variables and data was presented as median and interquartile ranges (IQR). Comparison were performed between HD, CAPD, CKD patients and controls using the Kruskal-Wallis test and a post-hoc analysis was performed using the Dunn’s test. Comparisons were made between those who had AsVD and those who did not, using the Wilcoxon rank-sum test.

Spearman correlation was used to determine the relationship between CIMT and cardiovascular risk factors among stage 3 CKD, CAPD, HD patients and the combination of the three groups. Multivariate regression analysis was performed to determine the relationship and contribution of cardiovascular risk factors to AsVD. The test of significance was taken as *p*-value < 0.05. Post regression analysis was done to determine the goodness of fit of the regression model.

## Results

The median age was 41 years (IQR 36.0–51.5) among stage 3 CKD patients, 39.5 years (IQR 35.0–46.5) among CAPD patients, and 40.5 years (36.0–49.0) among HD patients; Table 1. Increased WHR was most prevalent among CAPD patients. Hypertension was most prevalent among HD patients, occurring in 82.5% (*n* = 33/40) compared to 70% (*n* = 28/40) in CAPD patients and 17.1% (*n* = 7/41) among controls; (*p* < 0.001). Total cholesterol was elevated in 52.5% (*n* = 21/40) of CAPD patients compared to 22.5% (*n* = 9/40) of CKD patients and 12.2% (*n* = 5/41) controls, (*p* = 0.006 and < 0.001 respectively); however, total cholesterol levels were not elevated in HD patients. Although the rate of statin use was higher among CAPD patients (35%, 14/40) compared to the rate in HD patients, (17.5%, *n* = 7/40) there was no statistically significant difference, (x^2^ 3.164, p 0.075). Similarly, Angiotensin converting enzyme inhibitor (ACE-I) or Angiotensin receptor blocker (ARB) use was higher among PD patients compared to HD patients, however, not statistically significant, (x^2^ 0.621, p 0.432). Statin and ACE-I/ARB use were lowest among the stage 3 CKD patients. Atherosclerotic vascular disease was most prevalent among CAPD patients, occurring in 70% (*n* = 28/40) and showed significant differences compared to 47.5% (*n* = 19/40) among HD patients and 17.1% (*n* = 7/41) among controls; *p* = 0.041 and < 0.001 respectively; Table 1.

**Table 1:** Sociodemographic and clinical characteristics of the study population

Parameter	CKD (*n* = 40)	CAPD (*n* = 40)	HD (*n* = 40)	Controls (*n* = 41)
Age	41.0 (36.0–51.5)	39.5 (35.0–46.5)	40.5 (36.0–49.0)	41.0 (29.0–48.0)
DOD (years)		3.0 (1.5–4.0)	3.0 (2.0–6.5)	
Gender (n/%)				
Female	20 (50.0)	19 (47.5)	18 (45.0)	23 (56.1)
Male	20 (50.0)	21 (52.5)	22 (55.0)	18 (43.9)
BMI				
< 30 kg/m^2^	19 (47.5)	31 (77.5)	32 (80.0)	25 (61.0)
> 30 kg/m^2^	21 (52.5)	9 (22.5)	8 (20.0)	16 (39.0)
WHR				
Normal	12 (30.0)	9 (22.5)	15 (37.5)	28 (68.3)
Increased	28 (70.0)	31 (72.5)	25 (62.5)	13 (31.7)
Hypertension^a^				
Absent	15 (37.5)	12 (30)	17 (17.5)	34 (82.9)
Present	25 (62.5)	28 (70)	33 (82.5)	7 (17.1)
SCr (μmol/l)	124 (106–166)	1175 (878–1345)	513 (405–712	80 (63–89)
Albumin (g/l)	41.5 (38.5–44.0)	35.5 (33.0–40.0)	38.5 (35.0–41.0)	44.0 (42.0–45.0)
TC (mmol/l)				
< 5.17	31 (77.5)	19 (47.5)	40 (100)	36 (87.8)
> 5.17	9 (22.5)	21 (52.5)	0	5 (12.2)
LDL				
< 2.59	19 (47.5)	14 (35.0)	36 (90.0)	21 (51.2)
> 2.59	21 (52.5)	26 (65.0)	4 (10.0)	20 (48.8)
HDL^b^				
Low	30 (75.0)	21 (52.5)	15 (37.5)	23 (56.1)
Normal	10 (25.0)	19 (47.5)	25 (62.5)	18 (43.9)
SU (n/%)				
Yes	7 (17.5)	14 (35.0)	7 (17.5)	
No	33 (82.5)	26 (65.0)	33 (82.5)	
AAU (n/%)				
Yes	7 (17.5)	11 (27.5)	8 (20.0)	
No	33 (82.5)	29 (72.5)	32 (80.0)	
PBU (n/%)				
Yes	2 (5.0)	32 (80.0)	32 (80.0)	
No	38 (95.0)	8 (20.0)	8 (20.0)	
AsVD (n/%)				
Absent	21 (52.5)	12 (30.0)	21 (52.5)	34 (82.9)
Present	19 (47.5)	28 (70.0)	19 (47.5)	7 (17.1)

*CKD* Chronic kidney disease, *PD* Peritoneal dialysis, *HD* Haemodialysis, *DOD* Duration on dialysis, *BMI* Body mass index, *WHR* Waist-hip ratio, *TC* Total cholesterol, *LDL-C* Low density cholesterol, *HDL-C* High density cholesterol, *SU* Statin use, *AAU* ACE-I / ARB (angiotensin converting enzyme / angiotensin receptor blocker) use, *PBU* Phosphate binder use (calcium carbonate), *AsVD* Atherosclerotic vascular disease; ^a^Systolic blood pressure >  140 mmHg ± diastolic blood pressure > 90 mmHg, Low HDL-C = < 1.03 in male and < 1.29 mmol/l in female

All tested cardiovascular risk factors showed significant differences between the four groups using the Kruskal-Wallis test, except for age and Atherosclerotic Index of Plasma (AIP). The Dunn’s test result from a pairwise comparison showed that WHR, SBP, MABP, TC, LDL-C, non-HDL-C, LAD, EF, LVMI, CIMT, Castelli 1, AC, non-HDL-C were significantly increased among CAPD and HD patients compared to controls, while serum albumin and HDL-C were significantly decreased among CAPD and HD compared to controls, Table 2. Significantly higher serum phosphate levels and calcium-phosphate product (CaXPO_4_) were present in CAPD patients when compared with controls [1.6 (1.2–1.9) vs 1.0 (0.9–1.1), *p* < 0.001] and [40.1 (32.1–53.7) vs 29.5 (26.7–32.5), p < 0.001]; Table 2. Although serum levels of phosphate and CaXPO_4_ were higher among HD patients compared to controls, they were not statistically significant. 80% of CAPD and HD patients were on phosphate binders compared to 5% among the stage 3 CKD patients; the dialysate calcium was 1.75 mmol/l for CAPD and 1.5 mmol/l for HD patients.

**Table 2:** Comparison of clinical characteristics and echocardiographic parameters among chronic kidney disease, haemodialysis, peritoneal dialysis patients and controls

Parameter	K-W test (*n* = 161) x^2^ (*p*-value)	Dunn test (*p*-value)				
		CKD/controls	CAPD/controls	HD/controls	CKD/CAPD	CKD/HD
Age (years)	1.38 (0.71)					
BMI Kg/m^2^	16.3 (0.001)	0.077	0.312	0.086	0.001	0.001
WHR	20.6 (< 0.001)	0.001	< 0.001	0.020	0.758	0.310
SBP (mmHg)	39.1 (< 0.001)	< 0.001	< 0.001	< 0.001	0.263	0.025
MABP (mmHg)	89.1 (< 0.001)	< 0.001	< 0.001	< 0.001	0.073	0.007
TC (mmol/l)	50.6 (< 0.001)	0.019	0.005	< 0.001	0.007	0.001
TG (mmol/l)	14.0 (0.003)	0.427	0.570	< 0.001	0.820	0.005
LDL-C (mmol/l)	35.9 (< 0.001)	0.585	0.003	< 0.001	0.005	< 0.001
HDL-C (mmol/l)	10.6 (0.014)	0.001	< 0.001	< 0.001	0.592	0.152
Castelli1	12.4 (0.006)	0.966	0.030	0.436	0.008	0.256
Castelli2	10.9 (0.012)	0.261	0.249	0.085	0.011	0.373
AC	12.4 (0.006)	0.966	0.030	0.436	0.008	0.256
AIP	3.3 (0.354)					
non-HDL-C	39.8 (< 0.001)	0.308	< 0.001	0.001	0.008	< 0.001
eGFR (ml/min/1.73m^2^)	144.0 (< 0.001)	< 0.001				
Calcium (mmol/l)	8.3 (0.041)	0.974	0.034	0.072	0.033	0.145
Phosphate (mmol/l)	35.7 (< 0.001)	0.006	0.058	0.352	< 0.001	0.060
CaXPO_4_(mg^2^/dl^2^)	22.2 (< 0.001)	0.132	< 0.001	0.228	< 0.001	0.755
Albumin(g/l)	47.4 (< 0.001)	0.031	< 0.001	< 0.001	< 0.001	0.043
LAD	96.4 (< 0.001)	< 0.001	< 0.001	< 0.001	0.692	0.010
EF (%)	32.3 (< 0.001)	< 0.001	0.001	0.001	0.823	0.116
LVMI (g/m^2^)	24.2 (< 0.001)	0.136	< 0.001	< 0.001	0.027	0.034
FRS	13.1 (0.004)	0.035	0.005	0.913	0.178	0.074
Risk of CHD (%)	10.3 (0.017)	0.218	0.034	0.678	0.371	0.104
CIMT (mm)	36.3 (< 0.001)	< 0.001	< 0.001	< 0.001	0.012	0.673

*BMI* Body mass index, *WHR* Waist-hip ratio, *SBP* Systolic blood pressure, *MABP* Mean arterial blood pressure, *TC* Total cholesterol, *TG* Triglyceride, *LDL* Low density lipoprotein, *HDL* High density lipoprotein, *AC* Atherogenic coefficient, *AIP* Atherogenic index of plasma non-HDL – non high density lipoprotein eGFR – estimated glomerular filtration rate, *LAD* Left atrial diameter, *EF* Ejection fraction, *LVM* Left ventricular mass, *LVMI* Left ventricular mass index, *FRS* Framingham risk score; Risk of CHD – 10-year risk of coronary heart disease

**Fig. 1:**
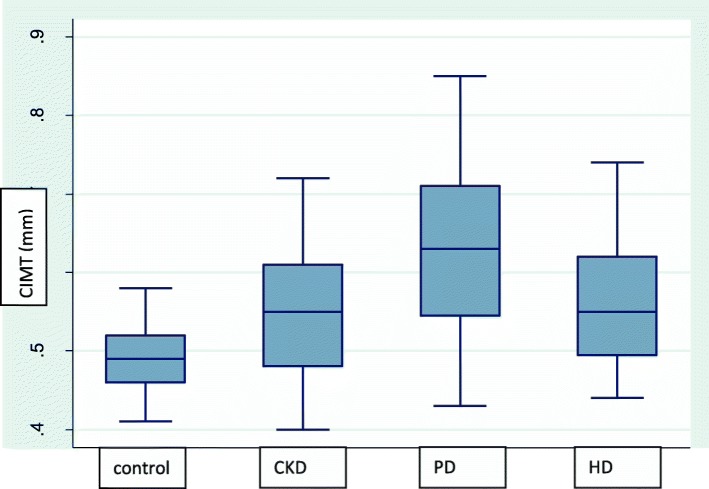
Carotid intima media thickness in controls, chronic kidney disease, continous ambulatory peritoneal dialysis and haemodialysis patients. CIMT – carotid intima media thickness; CKD – Chronic kidney disease KDOQI stage 3; HD – haemodialysis

Patients with AsVD were significantly older, and had increased WHR and LVMI, FRS and 10 year risk of developing CHD compared with those without AsVD. High density lipoprotein and left atrial diameter (LAD) showed a tendency towards significance when patients with AsVD were compared with patients without AsVD; Table 3. Higher values of CIMT and lower levels of serum albumin were seen in CAPD patients compared to the other study groups; Fig. 1.

**Table 3:** Comparison between those who had atherosclerotic vascular disease and those without atherosclerotic vascular disease among kidney disease patients

Risk factors	AsVD present (*n* = 66)	AsVD Absent (*n* = 54)	*p*-value
Age	43.5 (36.0–50.0)	37.0 (30.0–44.0)	0.002*
BMI	27.0 (22.5–31.9)	26.4 (22.0–30.5)	0.394
WHR	0.92 (0.88–0.96)	0.89 (0.85–0.94)	0.028*
SBP	148.5 (131.0–164.0)	147.0 (127–167)	0.411
MABP	139.8 (116.3–161.0)	141.8 (130.3–155.3)	0.792
TC	4.4 (3.4–5.3)	4.1 (3.5–4.7)	0.342
TG	1.2 (0.8–1.6)	1.3 (0.7–1.8)	0.444
LDL	2.5 (1.7–3.3)	2.3 (1.8–2.7)	0.150
HDL	1.2 (1.0–1.4)	1.1 (0.9–1.4)	0.065
Castelli1	3.5 (2.8–4.4)	3.6 (3.1–4.6)	0.560
Castelli2	1.9 (1.5–2.9)	2.0 (1.6–2.6)	0.914
AC	2.5 (1.8–3.4)	2.6 (2.1–3.6)	0.560
AIP	0.0 (−0.5–0.4)	0.1 (− 0.4–0.4)	0.202
non-HDL	3.2 (2.3–4.1)	3.0 (2.5–3.3)	0.518
LCI	9.5 (4.8–21.7)	10.1 (4.8–18.3)	0.868
EF	58.5 (53.0–64.0)	61.0 (56.0–64.0)	0.257
LAD	3.9 (3.5–4.5)	3.8 (3.2–4.2)	0.050
LVMI	122.9 (103.5–146.0)	98.6 (83.0–117.1)	< 0.001*
FRS	7.5 (3.0–11.0)	5 (0–9.0)	0.036
Risk of CHD (%)	2.0 (1.0–5.0)	0.8 (0.5–1.0)	< 0.001*

* - statistically significant, *p* < 0.05, *AsVD* Atherosclerotic vascular disease, *BMI* Body mass index, *WHR* Waist-hip ratio, *SBP* Systolic blood pressure, *MABP* Mean arterial blood pressure, *TC* Total cholesterol, *TG* Triglyceride, *LDL-C* Low density lipoprotein, *HDL-C* High density lipoprotein, *SCr* Serum creatinine, *eGFR* Estimated glomerular filtration rate, *CaXPO*_*4*_ Calcium-phosphate product, *AC* Atherogenic coefficient, *AIP* Atherogenic index of plasma, non-HDL-C- non- high density lipoprotein, *LCI* Lipoprotein combine index, *EF* Ejection fraction, *LAD* Left atrial diameter, *LVM* Left ventricular mass, *LVMI* Left ventricular mass index, *FRS* Framingham risk score, Risk of CHD – 10-year risk of coronary heart disease

CIMT correlated with age in all the kidney disease groups with the strongest association seen in CKD patients (r = 0.48, *p* = 0.002; Spearman correlation). A moderate positive correlation was observed between CIMT and LVMI across all kidney disease groups. There was a negative correlation between CIMT and Castelli indices 1 & 2 and AC among HD patients, (r = − 0.43, 0.006; r = − 0.44, *p* = 0.004; r = − 0.43, *p* = 0.006; respectively). There was no correlation between CIMT and lipoprotein ratios in stage 3 CKD and CAPD patients, and when all the kidney disease groups were combined; Table 4.

**Table 4:** Correlation between carotid intima media thickness and risk factors for cardiovascular disease among chronic kidney disease, peritoneal dialysis and haemodialysis patients

Parameter	CKD (n = 40) rho (*p*-value)	CAPD (*n* = 40) rho (p-value)	HD (*n* = 40) rho (p-value)	TOTAL (*n* = 120) rho (*p*-value)
Age	0.48 (0.002)	0.28 (0.077)	0.27 (0.099)	0.33 (< 0.001)
BMI	0.21 (0.205)	0.10 (0.538)	0.03 (0.845)	0.04 (0.651)
WHR	0.36 (0.023)	0.31 (0.051)	0.13 (0.429)	0.27 (0.003)
SBP	0.16 (0.318)	0.21 (0.187)	- 0.09 (0.579)	0.12 (0.187)
TC	- 0.05 (0.760)	- 0.04 (0.834)	- 0.13 (0.428)	0.08 (0.411)
TG	- 0.19 (0.240)	- 0.07 (0.681)	- 0.03 (0.845)	- 0.05 (0.606)
LDL	0.19 (0.244)	0.11 (0.501)	- 0.33 (0.040)	0.13 (0.173)
HDL	- 0.29 (0.069)	0.17 (0.291)	0.48 (0.002)	0.14 (0.130)
Castelli1	0.16 (0.322)	- 0.19 (0.238)	- 0.43 (0.006)	- 0.06 (0.500)
Castelli2	0.19 (0.245)	- 0.07 (0.662)	- 0.44 (0.004)	- 0.03 (0.772)
AC	0.16 (0.322)	- 0.19 (0.238)	- 0.43 (0.006)	- 0.06 (0.500)
AIP	- 0.01 (0.955)	- 0.14 (0.383)	- 0.20 (0.219)	- 0.09 (0.349)
non-HDL	0.09 (0.574)	- 0.11 (0.500)	- 0.29 (0.070)	0.04 (0.662)
LCI	0.04 (0.824)	- 0.10 (0.557)	- 0.20 (0.219)	0.01 (0.893)
Calcium (mmol/l)	- 0.12 (0.456)	- 0.02 (0.889)	- 0.09 (0.556)	- 0.12 (0.184)
Phos. (mmol/l)	0.00 (0.982)	0.09 (0.602)	- 0.13 (0.425)	0.10 (0.277)
CaXPo_4_ (mg^2^/dl^2^)	- 0.02 (0.898)	0.05 (0.749)	- 0.13 (0.425)	0.06 (0.508)
EF	- 0.19 (0.230)	- 0.01 (0.930)	0.01 (0.893)	- 0.11 (0.244)
LAD	0.20 (0.217)	0.55 (< 0.001)	- 0.17 (0.300)	0.21 (0.025)
LVMI	0.40 (0.012)	0.42 (0.006)	0.44 (0.005)	0.43 (< 0.001)

*CKD* Chronic kidney disease, *CAPD* Continuous ambulatory peritoneal dialysis, *HD* Haemodialysis, *BMI* Body mass index, *WHR* Waist-hip ratio, *SBP* Systolic blood pressure, *MABP* Mean arterial blood pressure, *TC* Total cholesterol, *TG* Triglyceride, *LDL* Low density lipoprotein, *HDL* High density lipoprotein, *eGFR* Estimated glomerular filtration rate, *AC* Atherogenic coefficient, *AIP* Atherogenic index of plasma; non-HDL- non- high density lipoprotein; *LCI* Lipoprotein combine index, *Phos* Phosphate, *CaXPo*_*4*_ Calcium-phosphate product, *EF* Ejection fraction, *LAD* Left atrial diameter, *LVM* Left ventricular mass, *LVMI* Left ventricular mass index

Binary logistic regression analysis showed that age and LVH were independent predictors of AsVD in dialysis patients after adjusting for gender, hypertension, TC and LDL-C and TG; Table 5.

**Table 5:** Relationship between atherosclerotic vascular disease and risk factors for cardiovascular disease among dialysis patients

Risk factors	OR	95% CI (*n* = 80)	*p*-value
Age (> 40 years)	3.11	1.00–9.65	0.049*
Gender	1.58	0.48–5.20	0.453
Hypertension^a^	1.49	0.37–5.97	0.575
LVH	49.79	2.58–959.68	0.010*
TC (> 5.17 mmol/l)	12.15	0.98–150.60	0.052
LDL-C (> 2.59 mmol/l)	1.64	0.34–7.99	0.541
TG (> 1.69 mmol/l)	0.69	0.17–2.80	0.599

* - Statistically significant, *p* < 0.05; LVH - left ventricular hypertrophy, *TC* Total cholesterol, *LDL-C* - low density lipoprotein, *BMI* Body mass index; ^a^systolic hypertension (systolic blood pressure > 140 mmHg)

## Discussion

Carotid intima media thickness is a reliable surrogate marker of subclinical atherosclerosis in the general population and increased CIMT has been associated with increased risk of cardiovascular events [20]. Similar findings have been reported among CKD and ESKD patients [21]. Our study showed a significantly higher prevalence of AsVD among CKD, CAPD and HD patients compared to controls. Almost three-quarters of the CAPD patients and almost half of the CKD and HD patients had AsVD compared to less than one-fifth of the control group. This is consistent with results from previous studies [8, 9]. The presence of AsVD in 17.1% of the controls in our study is comparable to the prevalence of 29.3% for peripheral arterial disease found among black rural dwellers in South Africa [22]; the lower prevalence observed in our study may be due to the exclusion of elderly people and diabetics from our study. Although the finding of high prevalence of AsVD in our stage 3 CKD and HD groups can be explained by the high prevalence of cardiovascular risk factors such as hypertension and reduced renal function among the kidney disease groups, we suggest that the very high prevalence of AsVD seen in CAPD may be associated with additional CAPD-related factors. Firstly, synergistic interplay between high glucose exposure and inflammation may contribute to the high prevalence of AsVD among CAPD patients [23, 24]. Exposure of CAPD patients to high glucose concentrations via the use of glucose-containing PD fluids, in the presence of high levels of inflammation, could result in increased glucose absorption and consequent hyperinsulinaemia, which has been associated with hypertension, obesity, dyslipidaemia and glucose intolerance [25]. In the setting of chronic hyperinsulinaemia, experimental evidence supports marked increase in lipogenesis in white adipose tissue and the liver [26]. From our study, the finding of higher prevalence of increased TC and LDL-C among CAPD patients compared to stage 3 CKD and HD patients further indicates the possibility of hyperinsulinaemia among PD patients. Secondly, we found significantly higher levels of uraemic retention solutes such as serum creatinine and urea among CAPD patients compared to HD and stage 3 CKD patients. In comparison to CAPD patients, the lower levels of uraemic retention solutes in HD patients, seen in our study may be explained by the effectiveness of current HD procedures in the clearance of uraemic toxins using biocompatible membranes, high flux dialyzers, dialysate flow rate of 500 ml/min and moderate blood flow rate of 350 ml/min, with dialysis undertaken thrice weekly and four hours per session. However, CAPD patients were dialysing at home and the prescribed dose of PD may not have been delivered due to incomplete adherence to treatment at home; a higher dialysis dose of PD than that delivered may have been required. In addition, the CAPD patients recruited into our study used conventional (glucose-based) PD fluids, which is acidic and contains supra-physiologic levels of lactate and a high glucose-induced osmolality of 395 mOsm/Kg H_2_0; this could have contributed to inflammation and worsening overall cardiovascular risk in this group of PD patients. Thirdly, higher uraemic retention solutes among CAPD patients may likely connote the presence of high levels of uraemic toxins such as middle molecules and protein-bound solutes among the CAPD patients which have been associated with CVD [27–29]. In our study, the median duration on CAPD was three years, therefore, loss of residual renal function in this group of patients, typically seen from three years after commencement of PD, could have contributed to the high levels of uraemic toxins. Bammens et al.^27^ found that increasing PD dose may compensate for declining residual renal function by increasing elimination of water soluble uraemic solutes but not middle molecules like p-cresol. Several studies have demonstrated an association between uraemic toxins (such as indoxyl sulphate, p-cresyl sulphate) and CVD [28, 29]. Both total and free indoxyl sulphate and p-cresyl sulphate were found to be independently associated with structural and functional markers of CVD [29]. Further studies on the relationship of chronic inflammation and uraemic toxins to AsVD in ESKD among black Africans will be required to establish this association. Serum levels of phosphate and calcium/phosphate product were higher among kidney disease patients with AsVD compared to patients without AsVD, but were not statistically significant, possibly because about 80% of ESKD patients were on phosphate binders and vitamin D.

Statistically significant differences were found in clinical and echocardiographic risk factors when each of the kidney disease groups was compared with controls. Of particular importance were systolic blood pressure (SBP), mean arterial blood pressure (MABP), WHR, non-HDL-C cholesterol, LAD, LVMI and CIMT which showed a near consistent *p* value of < 0.001 when the kidney disease groups were compared with controls. These findings were consistent with those documented in previous studies designed to evaluate the CVD risk factors in CKD [30, 31].

When patients who had AsVD were compared with those who did not, they were significantly older and had increased WHR and LVMI. The association between age and AsVD in our study is consistent with previous observations [32] and could be due to the fact that advancing age is associated with known cardiovascular risk factors such as hypertension, diabetes and vascular disease [33]. In addition, advancing age predisposes to endothelial dysfunction [34]. The finding of a significantly higher LVMI among patients who had AsVD and an association between AsVD and LVH among kidney disease patients is consistent with a previous study [35]. In addition to being categorised as a CVD, LVH has been shown as a cardiovascular risk factor in the general population [36] and in ESKD patients [37]. A significantly higher frequency of LVH was reported among black South African ESKD patients on HD compared to controls [8]. In the presence of hypertension and renal dysfunction, pressure and volume overload causes the cardiac myocytes to undergo conformational changes in order to compensate for haemodynamic alterations, resulting in LVH [38]. We found no significant difference in the 10-year risk of developing CHD when kidney disease patients were compared with controls, except in CAPD patients. Similarly, approximately 85% of kidney disease patients in each group were classified as low risk. These findings support the available evidence that the Framingham risk assessment for CHD has a poor predictive value for CHD among CKD patients [39]. In addition, the Framingham score does not consider the CKD-related risk factors for CVD, limiting its use in CKD patients [40]. On multiple comparisons between the four groups studied, significantly higher Framingham risk score and the 10-year risk of developing CHD were seen in CAPD patients when compared with controls and HD patients; these findings corroborates the higher carotid atherosclerotic burden seen in CAPD patients compared to HD patients in this study. The finding of significantly increased 10-year risk of developing CHD among kidney disease patients who had AsVD compared to those who do not have AsVD suggests that the Framingham tool may have a more useful discriminant role for CHD among CKD patients who have AsVD, compared to the predictive role for CHD seen in a non-CKD population. We recommend that a larger study will be helpful in confirming this finding.

Age (> 40 years) and LVH independently predicted AsVD among ESKD patients after adjusting for gender, WHR, systolic hypertension, LDL-C and TG. We found that LVH confers a 49-fold risk of AsVD among our ESKD patients. This highlights the robust relationship between LVH and AsVD among our ESKD group and underscores the importance of LVH as a CVD risk factor and the need to direct treatment strategies towards LVH reduction among CKD patients.

We recommend that longitudinal follow-up studies with a larger study population be designed to evaluate the relationship of AsVD to inflammation, hyperinsulinaemia and uraemic toxins, especially middle molecules, among black ESKD patients on CAPD and HD to ascertain the contribution of these risk factors to AsVD. We further recommend a randomised controlled trial among CAPD patients comparing CAPD patients using conventional PD fluids with patients using biocompatible PD fluids to determine the difference in CVD burden, if any, in both groups. However, while awaiting the outcomes from a randomised trial, we believe that patients using more biocompatible PD fluids may have a reduced CVD burden; therefore, it should be made more readily available and affordable.

This study has some limitations. While the study was adequately powered, the sample size is relatively small. We did not assess serum concentration of insulin and insulin resistance in these patients; these could have helped to confirm hyperinsulinaemia as a risk factor for AsVD among CAPD patients. The kt/V was also not documented, and this may have helped in objective assessment of adequacy of dialysis. The exclusion of the elderly, due to the relatively young age of the dialysis population at our centre, from this study could have reduced the overall prevalence of AsVD in the different patient groups investigated. Some of the patients recruited were on statins, this may have influenced the prevalence of AsVD reported in this study. The cross-sectional nature of this study allowed for measurements of the various parameters at a single point; a longitudinal study will provide data on the evolution of atherosclerosis over the period of CKD and dialysis.

## Conclusion

We found that AsVD was more prevalent among CAPD patients compared to pre-dialysis CKD and HD patients. Among all lipoprotein ratios assessed, non-HDL-C showed the most consistent significant difference between the groups. There was an association between AsVD and age, WHR and LVMI. Age (> 40 years) and presence of LVH independently predicted AsVD. The use of conventional PD fluids could have contributed to the higher prevalence of AsVD among CAPD patients; therefore, we advocate for improved accessibility and affordability of biocompatible PD fluids.

## Abbreviations

AI: Atherogenic index
AIP: Atherogenic index of plasma
AsVD: Atherosclerotic vascular disease
BMI: Body mass index
CAPD: Continuous ambulatory peritoneal dialysis
CIMT: Carotid intima media thickness
CKD: Chronic kidney disease
eGFR: Estimated glomerular filtration rate
ESKD: End-stage kidney disease
HD: Haemodialysis
HDL-C: High density lipoprotein
LDL-C: Low density lipoprotein
LVMI: Left ventricular mass index
non-HDL: Non-high density lipoprotein
PD: Peritoneal dialysis
SCr: Serum creatinine
TC: Total cholesterol
TG: Triglycerides
WHR: Waist-hip ratio
